# Spontaneous Generation

**Published:** 1851-04

**Authors:** 


### ANATOMY AND PHYSIOLOGY.

*Spontaneous Generation.*—M. Gros, of Heidelberg, has communicated to the Institute the substance of some curious researches relative to an animalcule which, according to the learned author, is developed spontaneously in the frog's bladder. The animalcule now alluded to has been denominated by M. Gros "*torquatina.*" It is produced by the mucous membrane of the bladder, which detaches a granule; this granule becomes the crown of the animalcule, while the neighboring vesicles become the body. To follow the development of the animalcule, it is necessary to take a small bit of the mucous membrane without injuring it, and then place it very cautiously under the microscope. Any pressure or solvent, even serum, will kill the animalcules. If these precautions be observed, the development of the animalcule may be followed. A mere unorganized granule is detached from the mucous membrane; this coalesces with other granules to form a certain portion of the animalcule; the latter then folds up its crown, fixes a number of vibratile cilia in various parts of its body, becomes oval, and constitutes an *opalina.* This latter forms a nidus in the mucosities of the intestine, deposits an egg, and the latter gives rise to a true worm of the ascaris kind.

If the above observations be correct, we must admit for certain entozoa a primitive species of generation different from any hitherto known—*Med. Times, Nov.* 16, 1850.

